# Chlorogenic Acid from Burdock Roots Ameliorates Oleic Acid-Induced Steatosis in HepG2 Cells through AMPK/ACC/CPT-1 Pathway

**DOI:** 10.3390/molecules28217257

**Published:** 2023-10-25

**Authors:** Kaiyang Ma, Weixi Sheng, Xinxin Song, Jiangfeng Song, Ying Li, Wuyang Huang, Yuanfa Liu

**Affiliations:** 1 College of Food Science and Technology, Nanjing Agricultural University, Nanjing 210095, China; makaiyang110@163.com (K.M.); songxin6809@163.com (X.S.); 2 Institute of Agro-product Processing, Jiangsu Academy of Agricultural Sciences, Nanjing 210014, China; songjiangfeng102@163.com (J.S.);; 3 Future Food (Bai Ma) Research Institute, Nanjing 211225, China

**Keywords:** chlorogenic acid, steatosis, β-oxidation of fatty acid, natural factor, molecular mechanism

## Abstract

Hepatic steatosis can cause liver dysfunction and cell injury, on which natural functional factors are expected to be an effective approach for long-term intervention. However, the cellular molecular mechanisms are unclear. Chlorogenic acid is a phenolic compound, which can regulate lipid metabolism and is abundant in burdock root. The aim of this study was to investigate the potential molecular mechanism of the effect of chlorogenic acid from burdock root (ACQA) on steatosis in HepG2 cells. In this study, we found that ACQA reduced the number of lipid droplets and lipid levels in oleic acid-treated HepG2 cells. Molecular mechanistic results showed that ACQA enhanced CPT-1 expression by activating AMPK-related signaling pathways, and the concentrations of Ca^2+^ and cAMP were increased with the intervention of ACQA. In addition, ACQA enhanced the β-oxidation of fatty acids, reduced alanine transaminase and aspartate transaminase, and inhibited apoptosis in oleic acid-treated HepG2 cells. Our studies elucidate a novel mechanism that ACQA enhances the β-oxidation of fatty acids through the AMPK/ACC/CPT-1 pathway to protect against steatosis in HepG2 cells, which provides insight into its molecular mechanism as well as intervention strategies for chlorogenic acid against fatty liver diseases.

## 1. Introduction

Hepatic steatosis takes the form of a malignant accumulation of triglycerides in liver cells, usually caused by eating disorders, which is common in chronic liver diseases of various etiologies. Hepatic steatosis eventually leads to liver cell injury and cirrhosis, which are also predictors of poor prognosis and mortality. Currently, severe hepatic steatosis requires long-term medical intervention, which may lead to frequent adverse reactions. The liver is the main site of lipid metabolism, and hepatic steatosis is accompanied by changes in the activities of key enzymes in lipid metabolism [1]. Fat accumulation in liver cells cannot be decomposed and metabolized in time when steatosis occurs, resulting in a decrease in the rate of decomposition of triglycerides (TG) into fatty acids or the β-oxidation of fatty acid [2]. However, steatosis is reversible [3], and natural factors in plants in particular have a better effect on hepatic steatosis [4].

Burdock (*Arctium lappa* L.) is a biennial plant of Compositae, which is a kind of medicinal and edible plant that is found in many countries and regions [5]. It has a variety of biological activities, and is traditionally used to regulate glycolipid metabolism, inflammation and constipation; this is due to its abundant secondary metabolites and beneficial natural factors [6]. In fact, our previous study found that chlorogenic acid, known as 5-O-caffeoylquinic acid (5-CQA), is the main phenolic in burdock root in different regions and during different picking periods [7]. In addition, our previous study found that ethanol extract from burdock root can not only reduce the body weight of rats, but also ameliorate hepatic steatosis in rats by increasing the rate of fatty acid β-oxidation in the liver of obese rats [8]. Therefore, chlorogenic acid in the ethanol extract of burdock root may be one of the key functional factors in ameliorating steatosis. In fact, the efficacy of various botanical drugs against liver disease is obvious, but it is usually complex with multiple signaling mechanisms, which makes them intervene in liver diseases with a variety of biological activities, such as their anti-inflammatory and anti-apoptotic roles [9]. Hence, it is necessary to explore the crucial molecular mechanism of chlorogenic acid from burdock root against fatty liver disease.

Chlorogenic acid is a well-known phenolic acid dietary factor, widely existing in plants. It has been found to ameliorate hepatic steatosis in mice [10], but the molecular mechanism remains unclear. Studies have reported that chlorogenic acid can phosphorylate adenosine monophosphate-activated protein kinase (AMPK) and activate AMPK-associated signaling pathways [11,12]. At the same time, hepatic phosphorylation levels of AMPK were reduced, and lipid metabolism was disordered in mice with fatty liver disease [13]. Phosphorylated AMPK activates a series of metabolism-related cascades that regulate the activation of key enzymes in lipid metabolism [14]. For instance, 3-hydroxy-3-methyl glutaryl coenzyme A reductase (HMG-CoR), glycerol-3-phosphate acyltransferases (GPAT) and sterol regulatory element-binding protein 1 (SREGBP-1) are related to lipid synthesis; hormone-sensitive lipase (HSL) promotes lipolysis; and acetyl-CoA carboxylase (ACC) and carnitine palmitoyltransferase-1 (CPT-1) are related to fatty acid β-oxidation, which are all regulated by AMPK. Our previous study also found that ethanol extract from burdock root can enhance the ability of fatty acid β-oxidation in the liver of obese rats by phosphorylating AMPK [8]. At the same time, AMPK is regulated by the second messenger such as cAMP and calcium/calmodulin-dependent protein kinase beta (Ca^2+^/CaMKK_2_); chlorogenic acid may affect the signaling pathway associated with the second messenger. Therefore, chlorogenic acid from burdock root may be one of the key functional factors to activate AMPK and regulate lipid metabolism.

Excessive development of steatosis eventually leads to the aggravation of fatty liver disease and a vicious cycle of obesity [15]. Currently, drugs targeting hepatic steatosis have a significant therapeutic effect, but the adverse reactions and side effects are also troubling. The search for natural factors to intervene in hepatic steatosis can reduce the toxicity of chemical synthetic drugs to the liver and kidney [16,17]. However, the molecular mechanism of natural functional factors against hepatic steatosis is always ambiguous, which limits its application. Therefore, we investigated the therapeutic effect of chlorogenic acid from burdock root on steatosis and its possible molecular mechanisms by establishing an oleic acid-induced steatosis model in HepG2 cells.

## 2. Results

### 2.1. ACQA Ameliorated Steatosis of HepG2 Cells

**Figure 1:**
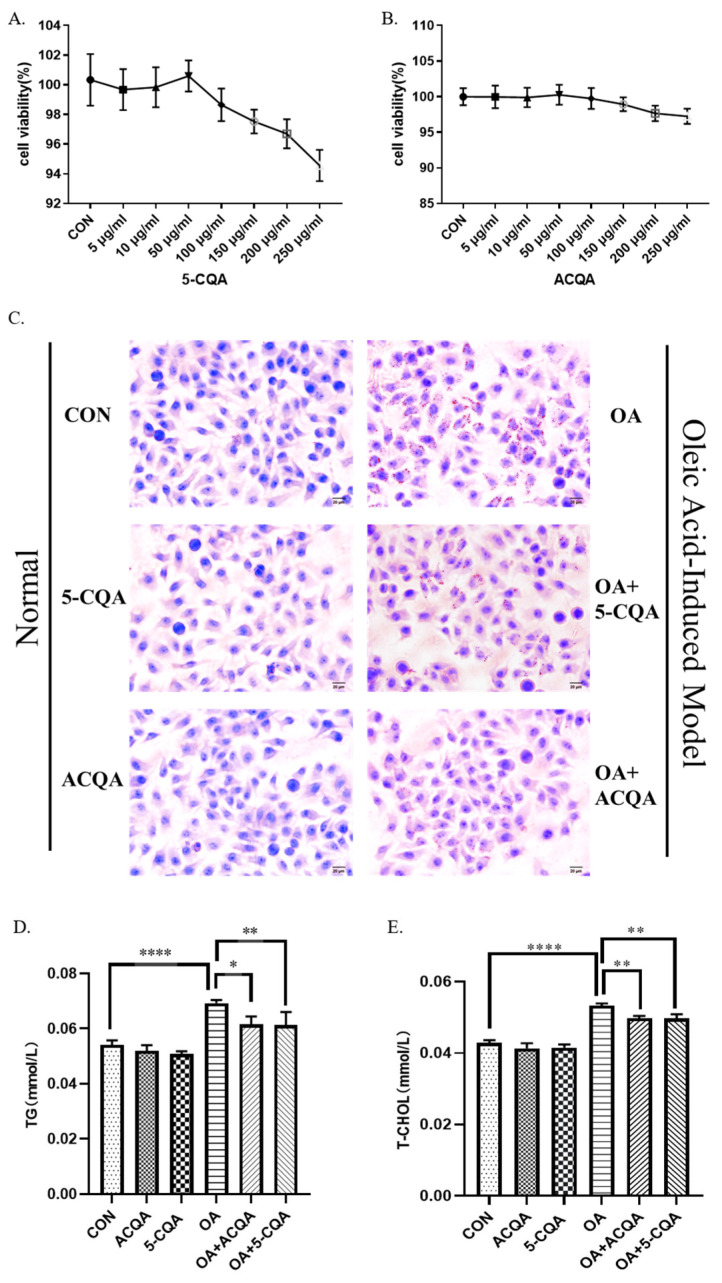
ACQA ameliorated steatosis of HepG2 cells. (**A**,**B**) The cell viabilities of HepG2 subjected to different concentrations of 5-CQA and ACQA were detected and analyzed via CCK-8 assay. The concentration of 50 µg/mL was the optimal concentration for both 5-CQA and ACQA. (**C**) Oil red O staining was performed in HepG2 cells. Red fat drops indicate the accumulation of triglycerides. Images presented are in ×40 magnification. Scale bar: 40× microscope is 20 μm. The levels of TG (**D**) and T-CHOL (**E**) were detected using assay kit and analyzed with enzyme-labeled apparatus. Data are expressed as mean ± SD, * *p* < 0.05, ** *p* < 0.01 and **** *p* < 0.0001, and compared with OA group, *n* = 3.

The optimal concentrations of 5-CQA and ACQA were obtained via CCK-8 assay. As shown in Figure 1A, the cell viability showed a downward trend with an increase in chlorogenic acid concentration from 5 μg/mL to 250 μg/mL, among which the highest cell viability was observed at 50 μg/mL. The results of chlorogenic acid from burdock root were similar to that of chlorogenic acid, and the cell viability 2s also reached the highest level at 50 ug/mL, as shown in Figure 1B. In addition, chlorogenic acid and chlorogenic acid from burdock root had no excessive proliferation effect on the HepG2 cells. A concentration of 50 μg/mL was selected as the optimal administration concentration of 5-CQA and ACQA for subsequent experiments. The steatosis model in HepG2 cells was obtained by 250 μM oleic acid combined with a high-glucose medium. Oil red staining showed that the number of lipid droplets in the OA group was significantly higher than that in the control group. Compared with the OA group, the number of lipid droplets was reduced in both intervention groups with 5-CQA and ACQA for 24 h, as shown in Figure 1C. In addition, the triglycerides (*p* < 0.05) and total cholesterol (T-CHOL) (*p* < 0.01) levels in the OA group were significantly higher than those in the control group. The levels of triglycerides and cholesterol in the OA + ACQA group and OA + 5-CQA group were significantly decreased (*p* < 0.01) compared with the model group, as shown in Figure 1D,E.

### 2.2. ACQA Affected the mRNA Levels of Key Enzymes Related to Lipid Metabolism

**Figure 2:**
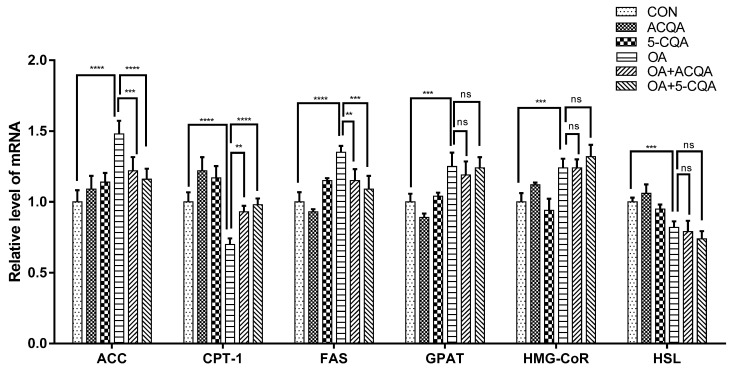
The mRNA levels of lipid metabolism-related genes in HepG2 cells. mRNA of each group was extracted, and mRNA levels of ACC, CPT-1, FAS, GPAT, HMG-CoR and HSL were detected via quantitative RT-PCR. Data are expressed as mean ± SD, ns *p* > 0.05, ** *p* < 0.01, *** *p* < 0.001 and **** *p* < 0.0001, and compared with OA group, *n* = 3.

Steatosis in the liver is often accompanied by the disorder of lipid metabolism; the key enzymes of lipid metabolism are especially affected [18]. To verify that oleic acid-induced steatosis in HepG2 cells is accompanied by changes in key enzymes of lipid metabolism, the mRNA levels of enzymes of lipid metabolism were detected using RT-qPCR, including acetyl-CoA carboxylase (ACC), carnitine palmitoyltransferase-1 (CPT-1), fatty acid synthase (FAS), glycerol-3-phosphate acyltransferases (GPAT), 3-hydroxy-3-methyl glutaryl coenzyme A reductase (HMG-CoR) and hormone-sensitive lipase (HSL). As shown in Figure 2, the mRNA levels of the above key enzymes in the OA group were significantly different from those in the control group (*p* < 0.001). However, the mRNA levels of ACC (*p* < 0.001) and FAS (*p* < 0.01) were significantly decreased in the OA + ACQA group and OA + 5-CQA group compared with the OA group. Meanwhile, the mRNA level of CPT-1 was significantly increased in the OA + ACQA group (*p* < 0.01) and OA + 5-CQA group (*p* < 0.0001) compared with the OA group.

### 2.3. ACQA Regulates ACC/CPT-1 Signaling Pathway through Phosphorylation of AMPK

**Figure 3:**
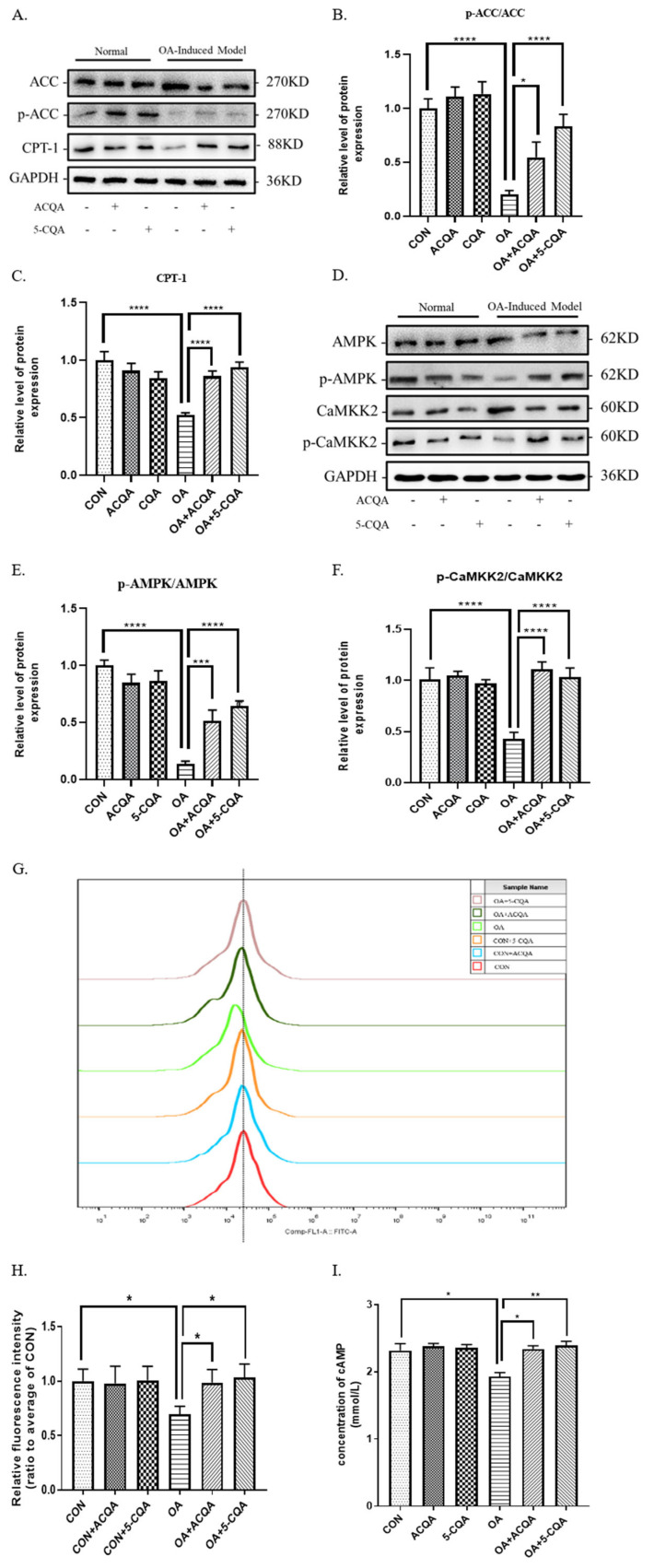
ACQA regulates ACC/CPT-1 pathway through the phosphorylation of AMPK. (**A**) The protein levels of ACC (including its phosphorylated levels) and CPT-1 were assessed via Western blot in HepG2. The relative levels of ACC (**B**) and CPT-1 (**C**) were analyzed. (**D**) The protein levels of AMPK, CaMKK_2_ and their phosphorylated levels were assessed through Western blot in HepG2. The relative phosphorylated levels of AMPK (**E**) and CaMKK_2_ (**F**) were analyzed. (**G**) Fluorescence intensity of calcium ion probe in HepG2 cells was detected with cell flow cytometer. The total detected cells of each group were 10,000. (**H**) Relative fluorescence intensity level was analyzed by ratio to the CON group and compared with the OA group. (**I**) The concentration of cAMP was detected and analyzed using enzyme-labeled apparatus. Data are expressed as mean ± SD, * *p* < 0.05, ** *p* < 0.01, *** *p* < 0.001 and **** *p* < 0.0001, and compared with OA group, *n* = 3.

As shown in Figure 3A–C, the levels of p-ACC/ACC in the OA group were reduced by nearly three times, and the protein expression of CPT-1 (*p* < 0.0001) was decreased by one time compared with the control group. However, the levels of p-ACC/ACC and CPT-1 (*p* < 0.0001) in the OA + ACQA group and OA + 5-CQA group were significantly increased compared with the OA group. These results indicate that ACQA and 5-CQA may regulate the protein expressions of ACC and CPT-1. As shown in Figure 3D–F, the ratio of *p*-AMPK/AMPK in the OA group was decreased by about one time compared with the control group, while the ratios of p-AMPK/AMPK in the ACQA and 5-CQA groups were significantly increased compared with the OA group, and nearly to the level of the control. In addition, the ratio of p-CaMKK_2_/CaMKK_2_ was significantly reduced by nearly one time (*p* < 0.0001) in the OA group compared with the control group, while the ratios of p-CaMKK_2_/CaMKK_2_ were significantly increased (*p* < 0.0001) in the OA + 5-CQA group and the OA + ACQA group compared to the OA group, and nearly to the level of the control. The fluorescence intensity of the Flou-3AM calcium ion probe was detected and analyzed using flow cytometry, as shown in Figure 3G,H. The peak of fluorescence intensity shifts to the left in the OA group compared with the CON group; this phenomenon disappeared after intervention with chlorogenic acid. Meanwhile, the relative fluorescence intensity level showed that the fluorescence intensity of the oleic acid-induced model group was significantly reduced compared with the CON group (*p* < 0.05), and the fluorescence intensity was significantly enhanced after chlorogenic acid intervention (*p* < 0.05). As shown in Figure 3I, the cAMP level of the OA group was significantly lower than that of the control group (*p* < 0.05), while the OA + 5-CQA group (*p* < 0.05) and OA + ACQA group (*p* < 0.01) significantly increased the cAMP level. These results suggest that both 5-CQA and ACQA phosphorylated AMPK through Ca^2+^/CaMKK_2_ and cAMP to regulate the protein expressions of p-ACC/ACC and CPT-1.

### 2.4. ACQA Enhanced Fatty Acid β-Oxidation and Reduced Cell Injury Caused by Steatosis in HepG2 Cells

**Figure 4:**
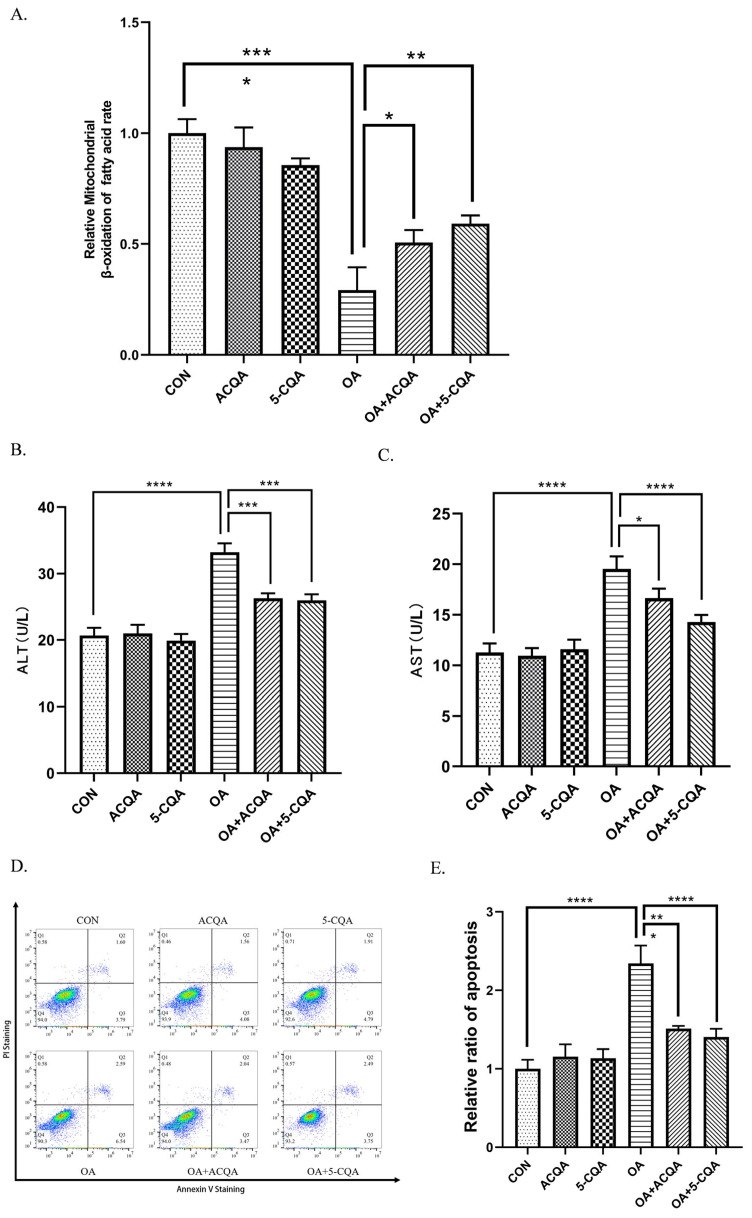
ACQA enhanced fatty acid β-oxidation and reduced injury caused by steatosis in HepG2 cells. (**A**) The rate of fatty acid β-oxidation in HepG2 cells was detected. The reduction rate of micromole ferricyanide was used to characterize the β-oxidation rate of fatty acids. (**B**,**C**) After the group was treated with chlorogenic acid, the culture medium was collected, and the ALT and AST levels in the culture medium were detected. (**D**) Apoptosis was assessed with annexin V/PI staining. (**E**) The data for analysis are expressed as the ratio of total apoptotic cells to the number of normal cells. Data are expressed as mean ± SD, * *p* < 0.05, ** *p* < 0.01, *** *p* < 0.001 and **** *p* < 0.0001, and compared with OA group, *n* = 3.

The ability of β-oxidation is expressed as the rate of reduction of micromolar ferric cyanide. In the results, as shown in Figure 4A, the ability of β-oxidation in the OA group (*p* < 0.01) was significantly lower than that in the control group, and the ability of β-oxidation in the model group was decreased by two times compared with the control group. In contrast, the abilities of β-oxidation in the OA + ACQA group (*p* < 0.05) and the OA + 5-CQA group (*p* < 0.01) were significantly increased compared with the OA group. These results indicate that both ACQA and 5-CQA inhibited the oleic acid-induced decrease in the ability of fatty acid β-oxidation. As shown in Figure 4B,C, the levels of alanine aminotransferase (ALT) and aspartate aminotransferase (AST) in the OA group were significantly higher than those in the control group (*p* < 0.0001), while the levels of ALT and AST in the OA + 5-CQA group and OA + ACQA group were significantly lower than those in the OA group. This indicates that 5-CQA and ACQA reduced OA-induced injury. The apoptosis level of HepG2 cells in the OA group was significantly higher than that in the control group (*p* < 0.0001); the level of apoptosis was increased by nearly 1.5 times. The intervention of ACQA (*p* < 0.001) and 5-CQA (*p* < 0.0001) may significantly inhibit the apoptosis of HepG2 cells, as shown in Figure 4D,E. These results indicate that ACQA and 5-CQA ameliorated steatosis, and protected cells from damage by enhancing fatty acid β-oxidation.

## 3. Discussion

Hepatic steatosis is characterized by an excessive accumulation of lipids in the liver cells. Eating disorders are a major cause of high fat intake. Normally, after the fat is absorbed and utilized by the tissues, excess fat is be stored in the adipose tissue or transported to the liver for metabolism. In particular, cholesterol and triglycerides are accompanied by apolipoprotein into the liver. The accumulation of fat aggravates the burden of lipid metabolism on the liver, which leads to fatty liver disease, and even causes a vicious cycle of obesity [19,20]. Among them, hepatic steatosis is a sign of lipid toxicity caused by an excessive accumulation of triglycerides in the liver cells. The occurrence and development of steatosis can lead to metabolic fatty liver disease. Studies have found that steatosis can be inhibited and ameliorated. Fibrates, such as fenofibrate, can inhibit steatosis by reducing the level of triglycerides in the liver [21], but treatment with this type of drug is often associated with serious adverse reactions, such as rhabdomyolysis, and cannot be used again for a long time. Recently, research has found that a variety of natural factors extracted from foods can protect the liver and reduce lipid levels [22,23]. For example, polyene phosphatidylcholine has been shown to have a good therapeutic effect and low adverse reactions in clinical use [24]. Fatty liver disease is often caused by chronic metabolic disease, which requires long-term prevention and treatment. To reduce the adverse reactions caused by long-term treatment, finding natural functional factors and foods to assist in the drug treatment of steatosis has gradually become an effective approach.

Our previous study found that ethanol extract from burdock root can ameliorate hepatic steatosis in rats and reduce the levels of triglycerides and cholesterol. Meanwhile, the polyphenols in the ethanol extract of burdock root are rich [25], which indicates that chlorogenic acid may be one of the functional factors to ameliorate the hepatic steatosis. Chlorogenic acid is a caffeoylquinic acid derivative, which can regulate lipid metabolism [26], but there are few reports on hepatic steatosis. Our results showed that chlorogenic acid and chlorogenic acid from burdock root could reduce the number of lipid droplets in HepG2 cells induced by oleic acid (Figure 1C), and ACQA had the same amelioration effect as 5-CQA. This was also demonstrated by changes in the triglyceride and cholesterol contents (Figure 1D,E), which also showed that the ability of ACQA to inhibit lipid toxicity was almost identical to that of 5-CQA. In addition, we observed that ACQA and 5-CQA did not have an excessive proliferation effect on HepG2 cells (Figure 1A). We believe that chlorogenic acid may not only ameliorate hepatic steatosis, but also not cause the proliferation of hepatic toxicity.

The potential mechanism of chlorogenic acid against hepatic steatosis is rarely studied. Steatosis is often accompanied by a disorder of lipid metabolism [27]. We considered that chlorogenic acid might regulate the key enzymes of lipid metabolism to ameliorate hepatic steatosis. We found that oleic acid could significantly affect mRNA levels of lipid metabolism-related enzymes in HepG2, and both 5-CQA and ACQA could inhibit the reduction in phosphorylation levels of ACC phosphorylation and enhance CPT-1 expression in HepG2 cells induced by oleic acid (Figure 2 and Figure 3A). It had been reported that chlorogenic acid from natural plants regulated lipid metabolism in high-fat diets fed to mice [10], and it was confirmed in this study. Interestingly, the protein expression levels of ACC and CPT-1 directly affected the metabolism of fatty acids. Normally, triglycerides transported to the liver are broken down into fatty acids and form acyl-coenzyme A, which is transferred to the mitochondria by CPT-1 [28]. The β-oxidation of fatty acids occurs in mitochondria, and breaks down the fatty acids into H_2_O and acetyl-CoA, which are involved in the tricarboxylic acid cycle to provide energy [29]. This is an important process in fat metabolism, and the ability of fatty acid β-oxidation affects triglyceride accumulation in the liver [30]. Under normal conditions, malonyl-CoA catalyzed by ACC inhibits the expression of CPT-1, while phosphorylation of ACC reduces this inhibition [31]. Thus, chlorogenic acid may strengthen the ability of fatty acid β-oxidation by enhancing the phosphorylation of ACC and CPT-1 protein expression. Our experimental results demonstrated this possibility (Figure 4A). Moreover, chlorogenic acid increased the β-oxidation of fatty acids in HepG2 stimulated by oleic acid, which was consistent with the trend of reduction in cytotoxicity. AMPK is known to regulate the expressions of key enzymes in glycolipid metabolism. The phosphorylation of ACC and protein expression of CPT-1 are also regulated by the phosphorylation of AMPK, which indicates that chlorogenic acid may play a regulatory role in AMPK activation. Our results demonstrated that ACQA and 5-CQA may enhance the phosphorylation level of AMPK in HepG2 cells stimulated by oleic acid (Figure 3D); therefore, we considered that chlorogenic acid could enhance the β-oxidation of fatty acid through the AMPK/ACC/CPT-1 signaling cascade, which is consistent with the action of polyphenols from apples in activating the AMPK signaling pathway [12]. The activation of AMPK itself is also regulated by various kinases and second messengers, especially Ca^2+^ /CaMKK_2_ and cAMP. Our results show that both 5-CQA and ACQA could enhance the concentrations of intracellular second messengers, calcium ions and cAMP (Figure 3G–I), and increase the phosphate level of CaMKK_2_ (Figure 3D). We believe that chlorogenic acid from burdock may activate Ca^2+^/CaMKK_2_ or cAMP to regulate the AMPK/ACC/CPT-1 pathway in HepG2 cells and strengthen the β-oxidation of fatty acids.

Excess lipid accumulation will inevitably inhibit cell activity and reduce the β-oxidation of fatty acid, which will eventually lead to hepatic steatosis and injury. As shown in Figure 4B,C, we detected an increase in ALT and AST in HepG2 stimulated by oleic acid, and this phenomenon was inhibited by 5-CQA and ACQA. In addition, chlorogenic acid also inhibited oleic acid-induced apoptosis of HepG2 cells (Figure 4D). This indicates that chlorogenic acid inhibited hepatic cell injury induced by oleic acid. Therefore, this result is consistent with burdock roots protecting hepatic cells against hepatic steatosis from the damage induced by lipotoxicity [8], which also indicates that chlorogenic acid is a critical functional factor in the treatment of metabolic fatty liver disease.

Although our study confirmed the efficacy of chlorogenic acid from burdock in steatosis and revealed its potential molecular mechanism in HepG2 cells, there is still a lack of animal studies to further support its pharmacological effects and applied dose on hepatic steatosis in vivo. Faced with these limitations of our study, we may conduct animal experiments to investigate the bioactivity of chlorogenic acid from burdock root against hepatic steatosis in the future, which will include its bioavailability and dose effects. Clinical trial results will also likely be an important indicator for evaluating the application of chlorogenic acid from burdock root in fatty liver disease. In addition, we noted that both of chlorogenic acid enhanced phosphorylation of AMPK in HepG2 cells, which indicated that it may activate upstream signaling molecules or certain targets. In fact, botanical drugs or functional factors usually rely on multiple targets to exert their biological activity; we will further explore the crucial targets of chlorogenic acid from burdock root against steatosis. We believe that it will provide an important theoretical basis for the application of botanical functional factors in fatty liver disease.

**Figure 5:**
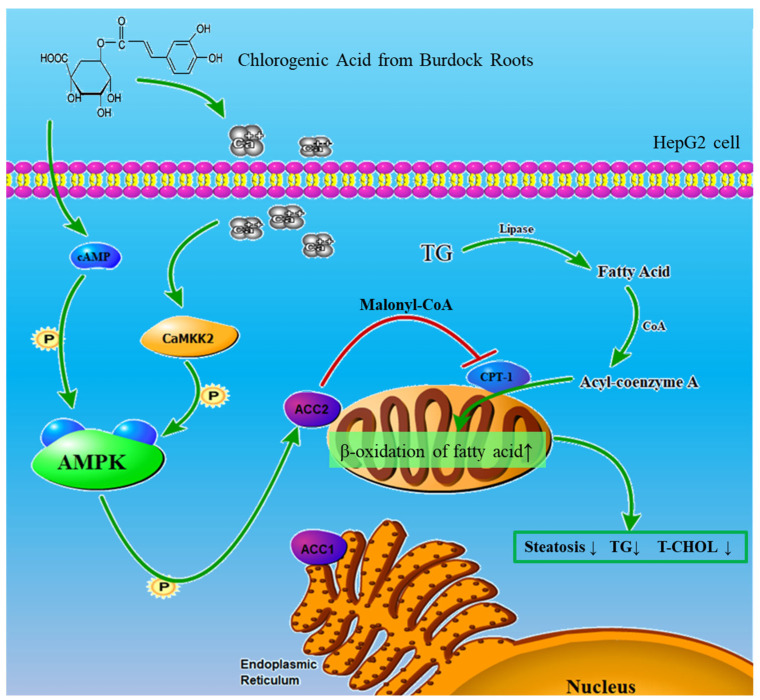
Chlorogenic acid from burdock roots ameliorates hepatic steatosis through AMPK/ACC/CPT-1 pathway in HepG2 cells. Triglycerides (TG) are broken down into fatty acids (FA) by the action of cellular lipase. Acyl-CoA formed by fatty acids and acetyl-CoA can be transported to mitochondria by CPT-1 for β-oxidation of fatty acids. Chlorogenic acid phosphorylates AMPK by increasing the concentration of cAMP and the Ca^2+^/CaMKK_2_ cascade reaction to enhance ACC phosphorylation levels and CPT-1 protein expression, which will strengthen β-oxidation of fatty acid and ameliorate steatosis.

In summary, our study further explored the molecular mechanism by which chlorogenic acid ameliorates hepatic steatosis, and confirmed that chlorogenic acid can enhance the β-oxidation of fatty acid through the AMPK/ACC/CPT-1 pathway to ameliorate oleic acid-induced steatosis of HepG2 cells (Figure 5). Chlorogenic acid from burdock roots can enhance the metabolic capacity of triglycerides, reduce fat buildup and protect hepatic cells from injury or lipid toxicity. These findings provide direction for finding natural factors from traditional plants that can be used as a long-term treatment for fatty liver disease. At the same time, our experimental results also clarified the cellular potential molecular mechanism of chlorogenic acid to ameliorate hepatic steatosis, and provided a theoretical basis for the application of natural intervention in chronic liver disease.

## 4. Materials and Methods

### 4.1. Reagents

The standard for chlorogenic acid (5-CQA, the purity is higher than 98%), the total cholesterol assay kit, triglyceride assay kit, alanine aminotransferase assay kit, aspartate aminotransferase assay kit and the mitochondria isolation kit were purchased from Nanjing Jiancheng Bioengineering Institute (Nanjing, China). A cell counting kit-8 (CCK-8) was purchased from Beyotime (Haimen, China). The oil red O stain kit (for cultured cells) was purchased from Beijing Solaibao Technology Co., LTD. (Beijing, China). Fluo-3AM was purchased from YeaSen (Shanghai, China). The annexin V-FITC/PI apoptosis detection kit, high-purity total RNA extraction kit, ChamQ Universal SYBR qPCR Master Mix for RT-PCR and a HiScript III RT Super Mix for qPCR (+gDNA wiper) were purchased from Vazyme Biotech (Nanjing, China). A fatty acid β-oxidation rate colorimetric test kit was purchased from GenMed (Cwmbran, UK). A human cyclic adenosine monophosphate (cAMP) ELISA kit was purchased from GenMed.

Primary antibodies against AMPK, phospho-AMPKα (Thr172), ACC and phosphor-ACC (Ser79) were purchased from Cell Signaling Technology (Danvers, MA, USA). Primary antibodies against CPT-1 and glyceraldehyde-3-phosphate dehydrogenase (GAPDH) were purchased from Proteintech (Sankt Leon-Rot, Germany). Horseradish peroxidase (HRP)-conjugated secondary antibodies were purchased from Cell Signaling Technology. The primary antibodies were used at 1:1000 dilutions, whereas 1:5000 dilutions were used for the secondary antibodies.

### 4.2. Preparation of Chlorogenic Acid from Burdock Root

*Arctium lappa* L. were harvested from Feng-county, Xuzhou city, China (30°55′00.00″ N 121°27′00.00″ E). Voucher specimens were identified by Dr. Fei Liu and deposited at the Xuzhou Polytechnic College of Bioengineering. Fresh burdock roots were sliced and dried in a constant oven temperature at 80 °C for 4 h. The slices were taken out and balanced at room temperature for 10 min, then the powder was ground with an XDW-DA ultra-micro pulverizer, screened using 80 mesh and stored at 4 °C. The burdock root powder was accurately weighed, and 60% ethanol was added according to the solid–liquid ratio of 1:30. Under the conditions of 70 °C and a power of 100 W, ultrasonic extraction was carried out for 2 h away from light. The extraction solution was centrifuged at 12,000 r/min through an H3-16KR table-top high-speed refrigerated centrifuge (HNKC, Changsha, China) for 20 min, and then vacuumized for filtration, repeated 3 times. Vacuum freeze-drying was performed at −80 °C after extraction. Then, the extract samples of were dissolved in ethanol (3 mg/mL, pH at 2) and dynamically adsorbed with AB-8 macroporous resin at a flow rate of 2 mL/min. The samples were eluted with 80 mL of 40% ethanol at a flow rate of 1 mL/min. Finally, the ethanol was fully volatilized to obtain chlorogenic acid from the burdock root (ACQA), which was dissolved in double-distilled water and used in the experiments. The purity of the chlorogenic acid from burdock root was 90.40%, based on the HPLC analysis. The HPLC profiles are presented in the Supplementary Materials, as shown in Figure S1.

### 4.3. Cell Cultures

HepG2 cells were obtained from the American Type Culture Collection (Manassas, VA, USA). The HepG2 cells were seeded on 120 mm Petri dishes and cultured with DMEM (GIBCO, Billings, MT, USA) containing 10% fetal bovine serum with 1% penicillin/streptomycin at 37 °C. After 24 h, the medium was changed every 3 days. When the bottom of the culture dish was 90% confluent, the cells were seeded in a six-well plate at a concentration of 1.5 × 10^5^ cells/mL for cultivation. When the bottom of the six-well plate was 90% confluent, the cells could be used for the experiments. The cell experiments were divided into a control group (CON group), model group (OA group) and intervention group with chlorogenic acid (5-CQA group) and chlorogenic acid from burdock root (ACQA group).

### 4.4. Cell Viability Assay

Five thousand HepG2 cells were seeded in 96-well plates and cultured for 24 h. After being treated with 10 μL of chlorogenic acid of different concentrations for 24 h, 10 μL of CCK-8 reagent was added to each well and incubated for 30 min in the dark. The CCK-8 reagent was reduced by an intracellular dehydrogenase to produce an orange precipitate, the color of which was proportional to the cell viability. The absorbance was measured at 450 nm to calculate the cell viability through an enzyme-labeled instrument.

### 4.5. Model of Oleic Acid-Induced Steatosis in HepG2 Cells

The steatosis model of HepG2 was established by the combination of a high glucose concentration and oleic acid. The HepG2 cells were treated with 4.5 g/L glucose and 250 μM OA for 24 h; the accumulation of lipid was obvious. Therefore, we chose the combination of 4.5 g/L glucose and 250 μM OA for 24 h to stimulate HepG2 cells for the steatosis model.

### 4.6. Oil Red O Staining of HepG2 Cells

HepG2 was seeded on glass coverslips (φ = 14 mm) and cultured for the experiment. The cells were treated for 24 h with chlorogenic acid after stimulation with oleic acid. The cell culture solution was discarded, and the cells were stained with oil red O for 10 min. After washing with PBS, hematoxylin was reverse stained for 30 s. The distribution of cell lipid droplets was observed at ×40 magnification with a NIKON ECLIPSE 80i advanced research microscope (NIKON, Tokyo, Japan).

### 4.7. RNA Reverse-Transcription and Quantitative RT-qPCR Analysis

The total cellular RNA was extracted with the high-purity total RNA extraction kit. The total RNA (1 μg) of each HepG2 sample was reverse transcribed into cDNA and amplified with ChamQ Universal SYBR qPCR Master Mix according to the manufacturer’s directions. The RT-PCR was performed with an ABI QuantStudio3 Real-Time PCR System (Applied Biosystems, Foster City, CA, USA) using HiScript III RT SuperMix for the qPCR (+gDNA wiper). After the addition of primers and template DNA to the master, the PCR thermal cycle parameters were as follows: 95 °C for 3 min, 40 cycles of 55 °C for 30 s and 95 °C for 15 s, with a melting curve from 60 to 95 °C to ensure amplification of a single product. In each sample, the β-actin was used as an endogenous control to normalize for differences in the amount of total RNA. The primer sequences are listed in Table 1.

### 4.8. Western Blot Analysis

The cells were lysed using RIPA lysate for protein extraction. The protein was extracted, and the protein concentration was measured with the total protein assay kit using the standard BCA method. The protein was isolated by sodium dodecyl sulfate polyacrylamide gel electrophoresis, and the proteins were transferred onto polyvinylidene fluoride (PVDF) membranes using EZ-Buffers C Western transfer buffer. After blocking with 10% milk for 1 h at 25 °C, the PVDF membrane was incubated with the various specific primary antibodies overnight at 4 °C. The antibodies were removed, and the membrane was washed with Tris-buffered saline with Tween 20 (TBST). Then, the membrane was incubated with the corresponding secondary antibody for 1 h at room temperature. The proteins were visualized and detected with an enhanced chemiluminescence Western blot assay reagent, and analyzed using the Image Quant™ LAS (G.E. Healthcare, Pittsburgh, PA, USA).

### 4.9. Apoptosis Detection of Cells by Flow Cytometry

The HepG2 cells were collected after treatment with 5-CQA for 24 h. Apoptosis of the cells was assessed by staining them with the annexin V-FITC/PI apoptosis detection kit, according to the manufacturer’s instructions. The apoptosis of cells was detected via flow cytometric analyses with the Guava easyCyte system 8 (Millipore, CA, USA).

### 4.10. Detection of Fatty Acid β-Oxidation Rate

The total protein was quantified using the Bradford protein assay kit after the cells were lysed with RIPA lysis buffer, and the mitochondria were extracted with the mitochondria isolation kit. After the mitochondrial separation solution was diluted to 10 mg/mL, it was repeatedly frozen and thawed three times to make it into mitochondrial fragments to enhance enzyme activity. The fatty acid β-oxidation capacity in HepG2 was detected via the fatty acid β-oxidation rate colorimetric test kit and analyzed with an automatic microplate reader (Epoch™, BioTek Instruments Inc., Winooski, VT, USA). Ferricyanide is reduced by carnitine during the β-oxidation of fatty acids, thus the rate of micromolar ferricyanide reduction can be used to evaluate the β-oxidation capacity.

### 4.11. Statistical Analysis

All of the data are presented as means ± SD. Statistical analyses were performed using Student’s *t*-test and ANOVA. In all of the studies, n indicates the number of samples per group, and cases in which *p* < 0.05 were considered statistically significant.

## Figures and Tables

**Table 1:** Sequences of quantitative PCR primers.

Target Gene	Primer	Sequence (5′-3′)
β-actin	Forward Reverse	CTCGCCTTTGCCGATCC CGCGGCGATATCATCATCC
ACC	Forward Reverse	ATGTCTGGCTTGCACCTAGTA CCCCAAAGCGAGTAACAAATTCT
CPT-1	Forward Reverse	ATGTCTGGCTTGCACCTAGTA CCCCAAAGCGAGTAACAAATTCT
FAS	Forward Reverse	AAGGACCTGTCTAGGTTTGATGC TGGCTTCATAGGTGACTTCCA
GPAT	Forward Reverse	CTGCTGAGTTGTCGCAATCG AACTCTTTCTCCGCTGGCTG
HMG-CoR	Forward Reverse	AGGTTCCAATGGCAACAACAGAAG ATGCTCCTTGAACACCTAGCATCT
HSL	Forward Reverse	TCAGTGTCTAGGTCAGACTGG AGGCTTCTGTTGGGTATTGGA

## Data Availability

All data included in this study are available upon request by contacting the corresponding author.

## Supplementary Materials

The following supporting information can be downloaded at: https://www.mdpi.com/article/10.3390/molecules28217257/s1, Figure S1: HPLC—profiles of chlorogenic acid and chlorogenic acid from burdock root.

## Sample Availability

Samples of the compounds are available from the authors.
