# Supplementary material for: Chlorogenic Acid from Burdock Roots Ameliorates Oleic Acid-Induced Steatosis in HepG2 Cells through AMPK/ACC/CPT-1 Pathway
Source: Molecules. 2023 Oct 25;28(21):7257. doi: 10.3390/molecules28217257 (PMC10647434; doi:10.3390/molecules28217257)
Supplement: Supplementary file 1 [file molecules-28-07257-s001.zip › molecules-2552385-supplementary.pdf]

Supplementary materials

The chlorogenic acid from burdock roots was qualitatively analyzed by comparing with standard of chlorogenic acid, and the concentration was obtained by HPLC. The sample concentration was 35.00 mg/ml. The concentration of chlorogenic acid in sample is 31.64 mg/ml, which was Calculated by establishing a standard curve. The purity of chlorogenic acid from burdock root is 90.40% based on HPLC analysis.

A.

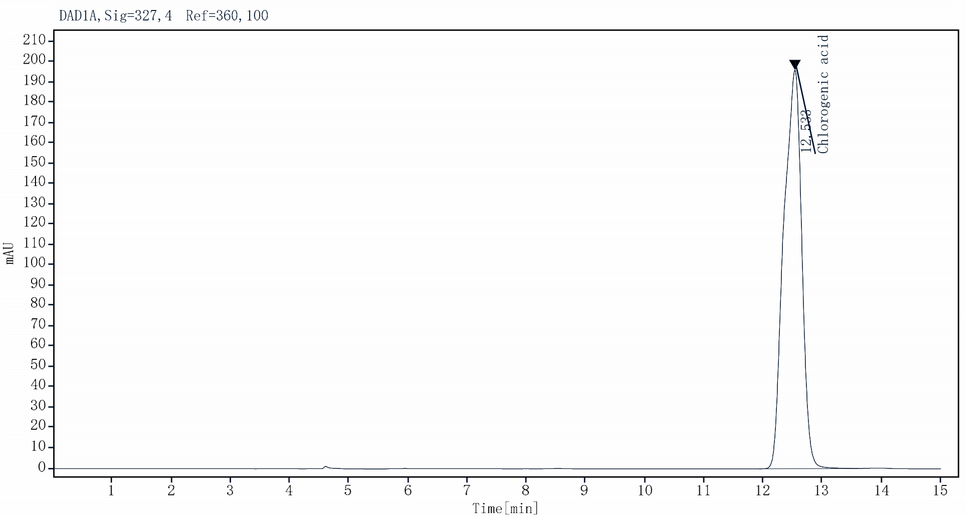

B.

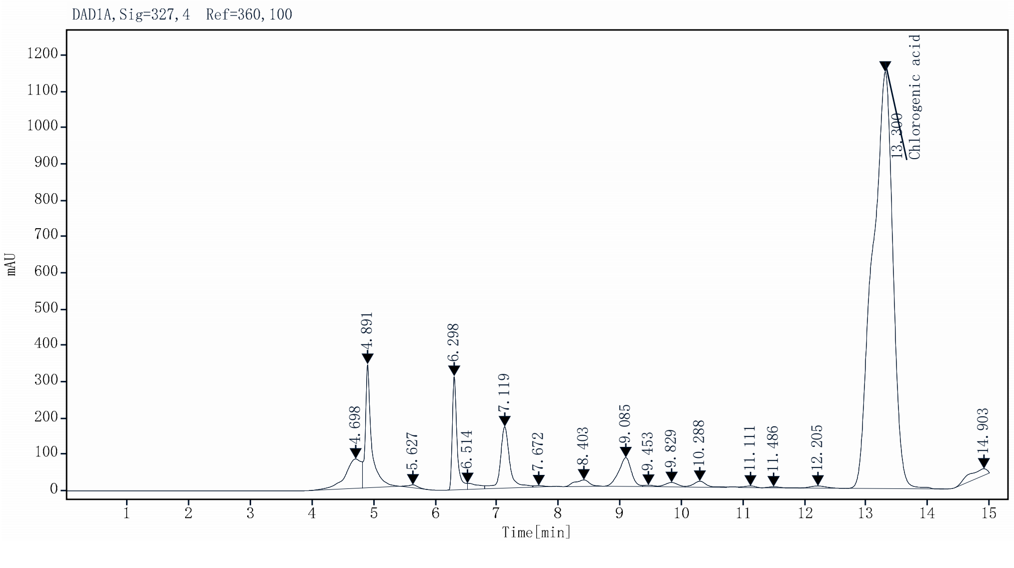

Figure S1. HPLC-profiles of chlorogenic acid and chlorogenic acid from burdock root

(A) chromatogram of standard of chlorogenic acid, and (B) chromatogram of chlorogenic acid from burdock root.
